# Epigenetic modifier-induced biosynthesis of novel fusaric acid derivatives in endophytic fungi from *Datura stramonium* L.

**DOI:** 10.1007/s13659-013-0010-2

**Published:** 2013-02-21

**Authors:** Han-Jing Chen, Takayoshi Awakawa, Jie-Yin Sun, Toshiyuki Wakimoto, Ikuro Abe

**Affiliations:** Graduate School of Pharmaceutical Sciences, The University of Tokyo, 7-3-1 Hongo, Bunkyo-ku, Tokyo, 113-0033 Japan

**Keywords:** endophyte, epigenetic modifier, biosynthesis, fusaric acid

## Abstract

**Abstract:**

The treatment of fungi with DNA methyltransferase (DNMT) and/or histone deacetylase (HDAC) inhibitors is a promising way to activate secondary metabolite biosynthetic pathways that are dormant under normal conditions. In this study, we included an HDAC inhibitor, suberoylanilide hydroxamic acid (SBHA), in the culture medium of endophytic fungi isolated from the medicinal plant *Datura stramonium* L. The production of two compounds was induced in the culture supplemented with SBHA, and their structures were determined to be the fusaric acid derivatives 5-butyl-6-oxo-1,6-dihydropyridine-2-carboxylic acid and 5-(but-9-enyl)-6-oxo-1,6-dihydropyridine-2-carboxylic acid. The result confirmed that the use of chemical epigenetic modifiers is an effective technique for promoting the expression of silent biosynthetic pathways to produce unique secondary metabolites.

**Graphical abstract:**

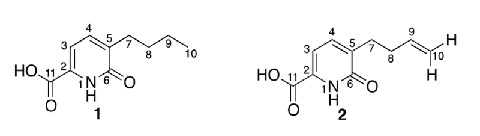

## Introduction

Fungi are talented organisms capable of producing pharmaceutically useful compounds, as exemplified by penicillin, cyclosporine and lovastatin.[1] The sequences of fungal genomes revealed the presence of many more genes encoding enzymes that produce secondary metabolites, as compared to those producing the known chemicals isolated from fungi. This suggested that many genes responsible for the production of secondary metabolites are not expressed in the artificial environment of the laboratory. Thus, the expression of such dormant genes in fungi is a promising way to obtain novel bioactive compounds. Recent studies revealed that histone deacetylase (HDAC) and DNA methyltransferase (DNMT) inhibitors are useful for activating dormant biosynthetic genes.[2–5] Indeed, the manipulation of the HDAC and DNMT activities by chemical agents alters the gene expression patterns in fungi, resulting in the production of novel secondary metabolites.[2–5]

Endophytic fungi from plants produce numerous biologically active compounds.[6] These also include compounds reported as originating from plants, such as taxol and camptothecin. These reports suggested that gene transfer events may occur between endophytic fungi and plants. Thus, investigations of secondary metabolite biosynthesis in endophytic fungi will broaden our comprehension of plant metabolite biosynthesis. *Datura stramonium* L. produces pharmaceutically important tropane alkaloids, such as scopolamine and hyoscyamine, via largely unknown biosynthetic pathways. To obtain clues about tropane alkaloid biosynthesis, we isolated several endophytic fungi from *D. stramonium* L, expecting to obtain fungal producers of tropane alkaloids.[7] Contrary to our expectations, none of the strains produced tropane alkaloids under the various conditions we tested. During this process, one strain isolated from the stem of *D. stramonium* L. produced several mycotoxins, such as alternariol, tenuazonic acid, and altertoxin II, in the presence of 5-azacytidine (a DNMT inhibitor) or suberoylanilide hydroxamic acid (SBHA, an HDAC inhibitor).[7] Likewise, another species was isolated from the root of *D. stramonium* L., which we named R1 and identified as *Fusarium oxysporum* f. sp. conglutinans strain MAFF744001, based on the sequence of its rRNA ITS region.[8] Since endophytic fungi are known as prolific sources of pharmacologically active compounds, we searched for the metabolites produced by R1. To fully exploit its biogenetic capability, we incubated the R1 strain in the presence of epigenetic modifiers, and monitored its products. Our approach led to the isolation of 5-butyl-6-oxo-1,6-dihydropyridine-2-carboxylic acid (**1**) and 5-(but-9-enyl)-6-oxo-1,6-dihydropyridine-2-carboxylic acid (**2**), which are derivatives of fusaric acid (Figure 1). **1** and **2** are novel compounds isolated from nature.

**Figure 1.** Structures of compounds **1** and **2**

*To whom correspondence should be addressed. E-mail: abei@mol.f.u-tokyo.ac.jp

**Results and Discussion**

The R1 strain was incubated in 75 mL of liquid medium in the presence and absence of 500 $\mu$M SBHA. The ethyl acetate extract of the culture filtrate was subjected to HPLC equipped with an ODS column. As a result, the production of the two new products **1** and **2**, which had not been detected under normal conditions, was induced by SBHA (Figure 2). In contrast, fusaric acid, which was identified by comparisons of its $^1$H and $^{13}$C NMR spectra with the published spectra (see Experimental Section), was detected in the cultures even in the absence of SBHA.

**Figure 2.** HPLC analyses of the culture supernatant of R1 strain: (i) a culture incubated in PD broth, (ii) a culture incubated in PD broth containing SBHA. Elutions were monitored by UV absorption at 220 nm.

To identify the structures of **1** and **2**, the ethyl acetate extract from a large-scale culture (6 × 75 mL) was separated by reverse-phase HPLC. The molecular formula of **1** was established as $C_{10}H_{13}NO_3$ by positive ion ESITOFMS, suggesting that the degree of unsaturation was five. The $^1$H NMR spectrum (DMSO-$d_6$) of **1** exhibited signals for two aromatic methine protons ($\delta_H$ = 6.81 and 7.31), three methylene protons ($\delta_H$ = 1.24, 1.43 and 2.39) and one methyl proton ($\delta_H$ = 0.84) (Table 1). The COSY analysis revealed that these aliphatic protons ($\delta_H$ = 1.24, 1.43 and 2.39) and one methyl proton ($\delta_H$ = 0.84) were in the same spin system, indicating that **1** contains an *n*-butyl group (Figure 3). The HMQC and HMBC analyses of **1** [$\delta_H$ = 2.39 (2H, t, $J$ = 7.5 Hz); $\delta_C$ = 138.2 and 163.1] confirmed the connection between the *n*-butyl group and the aromatic ring (Figure 3). In addition to these data, there were two quaternary carbons ($\delta_C$ = 162.2 and 163.1) in the $^{13}$C NMR spectrum of **1** (Table 2). Their chemical shifts were consistent with two carbonyl groups, and

**Figure 3.** COSY and HMBC correlations of **1** and **2**

**Table 1.** $^1$H NMR spectroscopic data of compound 1 and 2 in DMSO-$d_6$

| no. | 1[a] | 2[a] |
|---|---|---|
| 3 | 6.81 (1H, d, 7.0) | 6.84 (1H, d, 7.0) |
| 4 | 7.31 (1H, d, 7.0) | 7.34 (1H, d, 7.0) |
| 5 | | |
| 6 | | |
| 7 | 2.39 (2H, t, 7.5) | 2.46 (2H, m) |
| 8 | 1.43 (2H, m) | 2.25 (2H, m) |
| 9 | 1.24 (2H, m) | 5.77 (1H, m) |
| 10 | 0.84 (3H, t, 7.5) | |
| 10a | | 4.99 (1H, dd, 18.0, 1.75) |
| 10b | | 4.92 (1H, dd, 10.3, 1.75) |

[a]Measured at 500 MHz each must be a carboxyl and an amide carbonyl to satisfy the molecular formula. Thus, the structure of **1**, containing an alkyl group, aromatic ring, amide group and carboxyl group, was reminiscent of fusaric acid. In fact, the $^1$H and $^{13}$C NMR spectra of **1** were similar to those of fusaric acid, but lacked one of the three aromatic protons present in its $^1$H NMR spectrum [$\delta_H$ = 8.54 (1H, s)] (Tables 1 and 2), implying that **1** is a fusaric acid derivative with an oxygenated aromatic ring. The spin system of the aromatic protons of **1** suggested that a carbon atom (C-6) adjacent to a nitrogen atom in fusaric acid was oxygenated. Therefore, the structure of **1** was deduced to be 6-hydroxyl fusaric acid, which raised the question of whether **1** is stable as the pyridone or hydroxypyridine. Considering the fact that **1** was negative against the $FeCl_3$ reaction for the detection of phenols on TLC, the keto form of **1** might be the predominant tautomer. Therefore, we concluded that **1** is 5-butyl-6-oxo-1,6-dihydropyridine-2-carboxylic acid (Figure 1).

**Table 2.** $^{13}$C NMR spectroscopic data of compound 1 and 2 in DMSO-$d_6$

| no. | 1[a] | 2[a] | no. | 1[a] | 2[a] |
|---|---|---|---|---|---|
| 2 | 134.9 C | 135.7 C | 8 | 30.3 $CH_2$ | 32.5 |
| 3 | 109.3 CH | 110.3 CH | 9 | 22.5 $CH_2$ | 138.8 CH |
| 4 | 136.6 CH | 137.7 CH | 10 | 14.3 $CH_3$ | |
| 5 | 138.2 C | 139.0 C | 10a | | 116.1 $CH_2$ |
| 6 | 163.1 C | 162.6 C | 10b | | 116.1 $CH_2$ |
| 7 | 30.2 $CH_2$ | 30.5 $CH_2$ | 11 | 162.2 C | 162.4 C |

[a]Measured at 125 MHz

The $^1$H NMR spectra of **2** were quite similar to those of **1**, except for three proton signals ($\delta_H$ = 4.92, 4.99 and 5.77) (Table 1). The COSY analysis revealed that these protons ($\delta_H$ = 4.92, 4.99 and 5.77) and the aliphatic protons ($\delta_H$ = 2.25, and 2.46) were in the same spin system, indicating that **2** contains a 9,10-dehydrobutyl group in place of the *n*-butyl group of **1** (Figure 3). In a similar manner as for **1**, the HMQC and HMBC analyses of **2** [$\delta_H$ = 2.46 (2H, m); $\delta_C$ = 137.7 and 162.6] confirmed the connection between the 9,10-dehydrobutyl group and the aromatic ring. Therefore, **2** was assumed to be the dehydro form of **1**. The methyl ester of **2** generated an ion peak corresponding to the $[M + H]^+$ at $m/z$ 208.0956 (calcd for $C_{11}H_{14}NO_3$ 208.0968) in ESITOFMS. These data revealed that the structure of **2** is 5-(but-9-enyl)-6-oxo-1,6-dihydropyridine-2-carboxylic acid (Figure 1).

The antibacterial activities of **1** and **2** were tested with *Bacillus cereus*. Compounds **1** and **2** were dissolved in MeOH and serially diluted to final sample concentrations from 400–0

$\mu$g/mL. While fusaric acid exhibited antibacterial activity (MIC = 25 $\mu$g/mL), neither **1** nor **2** showed any activity, indicating that the oxidation of the pyridine ring significantly diminished the antibacterial activity of fusaric acid. This fact implied that these compounds would not kill bacteria within or on the plant, and thus not confer any advantage for the survival of its producer. Since fusaric acid is toxic to plant physiological processes as well as to microorganisms,[9] it might be oxidized into **1** and **2** for the sake of alleviating the toxicity to the plant. This led us to assume that the biosynthetic routes for the production of **1** and **2** from fusaric acid could be specific for endophytic *Fusarium* species. In fact, *F. oxysporum* FGSC9935, which is not an endophytic strain, could not produce **1** and **2** even in the presence of SBHA **(**data not shown**)**. Alternatively, compounds **1** and **2** are biosynthetic precursors of fusaric acid. A reduction step followed by a dehydration step would convert **1** into fusaric acid.

By supplying SBHA to the culture, 5-butyl-6-oxo-1,6-dihydropyridine-2-carboxylic acid (**1**) and 5-(but-9-enyl)-6-oxo-1,6-dihydropyridine-2-carboxylic acid (**2**) were isolated from the endophyte strain belonging to *F. oxysporum*. Even though fusaric acid was previously isolated from *F. oxysporum*[10] and *F. verticillioides*[11], both **1** and **2** were novel compounds isolated from natural sources. The synthesis of **1** was outlined in a US patent reported in 1975, in which a chemical library of 5-alkylpyridine-2-carboxylates was constructed to obtain compounds with dopamine-$\beta$-hydroxylase activity.[12] In this patent, neither the NMR spectra nor the dopamine-$\beta$-hydroxylase activity of **1** were described. Therefore, this is the first report of the spectral data of **1**. On the other hand, compound **2** has not been synthesized or isolated so far. Hence, this novel compound was not detected until an epigenetic modifier was used to treat the fungal culture. This report demonstrates that the use of epigenetic modifiers is extremely effective for isolating novel compounds from nature.

## Experimental Section

**General Experimental Procedures.** *D. stramonium* L. was a kind gift from Dr. Noguchi, of the University of Shizuoka. SBHA was purchased from Sigma. PD broth was purchased from Difco. Oligonucleotide primers were purchased from Operon Biotechnologies (Tokyo).

**Isolation and Identification of Endophytic Fungi.** The isolation of endophytic fungi from *D. stramonium* L. was performed as described previously.[7] The R1 strain was identified by a DNA fragment of the fungal rDNA ITS region.[8] The genomic DNA of R1 was extracted. PCR was performed with the primers ITR1 (5'-TCCGTAGGTGAACCTGCGG-3') and ITS4 (5'-TCCTCCGCTTATTGATATGC-3') and DNA isolated from R1 by phenol/chloroform/isoamyl alcohol = 25:24:1 extraction, using KAPATaq Extra DNA polymerase (KAPA Biosystems) and the following program: 94 ºC, 2 min; (94 ºC, 15 s; 55 ºC, 15 s; 72 ºC, 60 s) × 35 cycles. The amplified fragment was sequenced on an Applied Biosystems 3130XL sequencer.

**HPLC Analysis of the Compound Accumulated in the Broth of R1.** The mycelia of R1 were grown on PD agar medium at 30 ºC for 7 days and then inoculated into 75 mL of liquid PD medium containing 500 $\mu$M SBHA in a 500 mL flask, incubated at 28 ºC under static conditions for 7 days. The broth culture filtrate was collected and successively extracted with ethyl acetate. The organic layer was concentrated and subjected to HPLC analysis. The extract was analyzed by HPLC equipped with a TSKgel ODS-80Ts QA column (TOSO), with a solvent system of water containing 0.1% TFA (solvent A) and methanol containing 0.1% TFA (solvent B), at a flow rate of 0.8 mL/min and a column temperature of 40 ºC. Separation was performed with a linear gradient from solvent B/solvent A (10:90) to 100:0 within the following 30 min, 100:0 for an additional 15 min and a linear gradient from 100:0 to 10:90 within the following 5 min.

**Characterization of 1 and 2.** The R1 strain was inoculated into six 500 mL flasks, each with 75 mL of liquid PD medium containing 500 $\mu$M SBHA, and incubated at 28 ºC for 7 days. The broth culture filtrate was collected, successively extracted with ethyl acetate and then dried, for detection by HPLC. For structural identification, the crude extract was separated by HPLC equipped with a COSMOSIL 5C18-AR-II column (Nacalai Tesque), using a solvent system of water containing 0.1% TFA (solvent A) and methanol containing 0.1% TFA (solvent B), at a flow rate of 2.5 mL/min and a column temperature of 40 ºC. The gradient program was the same as described above. As a result, 5.28 mg of **1**, 1.23 mg of **2** and 4.32 mg of fusaric acid were prepared. The $^1$H and $^{13}$C NMR spectra were recorded on a JEOL ECX-500 spectrometer in DMSO-$d_6$. The NMR data of **1** and **2** are summarized in Tables 1 and 2, and the NMR data of fusaric acid are summarized below. **1** was subjected to an ESITOFMS (BRUKER DALTONICS) analysis, and the $[M + H]^+$ ion peak at *m/z* 196.0989 $[M + H]^+$ (calcd for $C_{10}H_{14}NO_3$, 196.0968) was detected.

**Preparation of Methyl Ester of 2.** To the MeOH solution (0.1 mL) of **2** (0.1 mg) was added 600 $\mu$L of a diethyl ether solution of diazomethane. The mixture was allowed to slowly warm to room temperature. After 6 hours, the sample was subjected to the ESITOFMS analysis, and the $[M + H]^+$ ion peak at *m/z* 208.0956 (calcd for $C_{11}H_{14}NO_3$ 208.0968) was detected.

**Antibacterial Activity Assay.** The antibacterial activity of the test compounds was assessed using *Bacillus cereus*, which was grown in LB medium at 37 ºC for 12 hours. The assays were conducted in 96-well microtiter plates. A 10 $\mu$L portion of the LB medium culture of *B. cereus* was suspended in 10 mL Mueller Hinton broth, and an appropriate volume was added to each row. The test compounds were dissolved in MeOH, and 2 $\mu$L aliquots were added to the first row. Serial dilutions were prepared to make final sample concentrations of 400, 200, 100, 50, 25, 12.5, 6.25, 3.13, 1.56, 0.78, 0.39, and 0 $\mu$g/mL, and 2 $\mu$L aliquots were added to the following rows. After an incubation at 37 ºC for 16 hours, the MIC values were determined visually.

**5-Butyl-6-oxo-1,6-dihydropyridine-2-carboxylic acid (1):** white solid; UV (MeOH) $\lambda_{max}$ 247, 311 nm; $^1$H NMR (DMSO-

$d_6$, 500 MHz) and $^{13}$C NMR (DMSO-$d_6$, 125 MHz) data, see Tables 1 and 2; ESIMS (positive) *m/z* 196 $[M + H]^+$; HRESIMS *m/z* 196.0989 (calcd for $C_{10}H_{14}NO_3$, 196.0968).

**5-(But-9-enyl)-6-oxo-1,6-dihydropyridine-2-carboxylic acid (2):** white solid; $UV_{max}$ (MeOH) $\lambda_{max}$ 247, 311 nm; $^1$H NMR (DMSO-$d_6$, 500 MHz) and $^{13}$C NMR (DMSO-$d_6$, 125 MHz) data, see Tables 1 and 2; ESIMS (positive, methylated **2**) *m/z* 196 $[M + H]^+$; HRESIMS *m/z* 208.0956 (calcd for $C_{11}H_{14}NO_3$ 208.0968).

**Fusaric acid:** white solid; $UV_{max}$ (MeOH) $\lambda_{max}$ 224, 269 nm. $^1$H NMR (DMSO-$d_6$, 500 MHz): $\delta$ 0.92 (3H, m, H-10), 1.32 (2H, m, $J$ = 1.7 Hz, H-9), 1.58 (2H, m, $J$ = 2.3 Hz, H-8), 2.66 (2H, t, $J$ = 8.0 Hz, H-7), 7.85 (1H, d, $J$ = 8.0 Hz, H-4), 8.00 (1H, d, $J$ = 8.0 Hz, H-3), 8.54 (1H, s, H-6). $^{13}$C NMR (DMSO-$d_6$, 125 MHz): $\delta$ 14.3 ($CH_3$, C-10), 22.3 ($CH_2$, C-9), 32.3 ($CH_2$, C-7), 33.1 ($CH_2$, C-8), 125.2 (CH, C-3), 137.7 (CH, C-4), 142.5 (C, C-5), 146.2 (C, C-2), 149.7 (CH, C-6), 166.5 (C, C-11). ESIMS (positive) *m/z* 180 $[M + H]^+$; HRESIMS *m/z* 180.1030 $[M + H]^+$ (calcd for $C_{10}H_{14}NO_2$, 180.1019).

## Acknowledgments

This work was supported by Grants-in-Aid for Scientific Research from the Ministry of Education, Culture, Sports, Science and Technology, Japan.

**Open Access** This article is distributed under the terms of the Creative Commons Attribution License which permits any use, distribution, and reproduction in any medium, provided the original author(s) and source are credited.

## References

[1] Keller N P, Turner G, Bennett J W (2005). Nat. Rev. Microbiol..

[2] Williams R B, Henrikson J C, Hoover A R, Lee A E, Cichewicz R H (2008). Org. Biomol. Chem..

[3] Henrikson J C, Hoover A R, Joyner P M, Cichewicz R H (2009). Org. Biomol. Chem..

[4] Wang X, Sena Filho J G, Hoover A R, King J B, Ellis T K, Powell D R, Cichewicz R H (2010). J. Nat. Prod..

[5] Asai T, Yamamoto T, Oshima Y (2011). Tetrahedron Lett..

[6] Kharwar R N, Mishra A, Gond S K, Stierle A, Stierle D (2011). Nat. Prod. Rep..

[7] Sun J, Awakawa T, Noguchi H, Abe I (2012). Bioorg. Med. Chem. Lett..

[8] Burmeister H R, Grove M D, Peterson R E, Weisleder D, Plattner R D (1985). Appl. Environ. Microbiol..

[9] Wu H S, Yin X M, Liu D Y, Ling N, Bao W, Ying R R, Zhu Y Y, Guo S W, Shen Q R (2008). Plant. Soil..

[10] Bacon C W, Porter J K, Norred W P, Leslie J F (1996). Appl. Environ. Microbiol..

[11] Xie Y, Zhang W, Li Y, Wang M, Cerny R L, Shen Y, Du L (2011). Mycology.

[12] Kühnis, H.; Egil, C.; Eichenbuerger, K.; Hedwall, P. R. U.S. Patent 3,914,239, 1975.

